# Transfer Discriminative Dictionary Pair Learning Approach for Across-Subject EEG Emotion Classification

**DOI:** 10.3389/fpsyg.2022.899983

**Published:** 2022-05-10

**Authors:** Yang Ruan, Mengyun Du, Tongguang Ni

**Affiliations:** ^1^HUA LOOKENG Honors College, Changzhou University, Changzhou, China; ^2^School of Computer Science and Artificial Intelligence, Changzhou University, Changzhou, China

**Keywords:** electroencephalogram signals, transfer learning, dictionary pair learning, emotion classification, across-subject

## Abstract

Electroencephalogram (EEG) signals are not easily camouflaged, portable, and noninvasive. It is widely used in emotion recognition. However, due to the existence of individual differences, there will be certain differences in the data distribution of EEG signals in the same emotional state of different subjects. To obtain a model that performs well in classifying new subjects, traditional emotion recognition approaches need to collect a large number of labeled data of new subjects, which is often unrealistic. In this study, a transfer discriminative dictionary pair learning (TDDPL) approach is proposed for across-subject EEG emotion classification. The TDDPL approach projects data from different subjects into the domain-invariant subspace, and builds a transfer dictionary pair learning based on the maximum mean discrepancy (MMD) strategy. In the subspace, TDDPL learns shared synthesis and analysis dictionaries to build a bridge of discriminative knowledge from source domain (SD) to target domain (TD). By minimizing the reconstruction error and the inter-class separation term for each sub-dictionary, the learned synthesis dictionary is discriminative and the learned low-rank coding is sparse. Finally, a discriminative classifier in the TD is constructed on the classifier parameter, analysis dictionary and projection matrix, without the calculation of coding coefficients. The effectiveness of the TDDPL approach is verified on SEED and SEED IV datasets.

## Introduction

Emotion is an advanced cognitive activity of human beings and plays an increasingly important role in human rational thinking, decision-making, perception and learning. Since human emotional state is a complex psychological and physiological process, the study of human emotion is a complex cognitive process. At present, emotion classification is an important research direction in emotional computing and artificial intelligence. Emotion classification has been widely used in intelligent transportation, distance education, and human-computer interaction. However, as an interdisciplinary research field, there are still many challenges in emotion classification based on artificial intelligence technology. Early researchers usually used facial expressions, audio and peripheral physiological signals (body temperature, blood pressure, pulse, respiration, etc.) to indirectly study emotions (Shu et al., 2018). However, such information is easy to disguise and not objective, it is easy for users to deceive the detection device by subjectively controlling the external expression of emotions, making it difficult to objectively and accurately describe the user's emotional state. With the development of cognitive science, the relationship between the specific location of the cerebral cortex and emotion is gradually recognized, so it is a good choice to directly study the activity of the cerebral cortex to study the emotional state. Researchers can use the existing knowledge of signal processing to directly process physiological signal data, which also greatly promotes the development of emotion recognition. Studies have shown that EEG signals can be used as an informative feature of emotional state. EEG signals have the advantages of being difficult to camouflage, portable and non-invasive acquisition, high temporal resolution, and can directly reflect brain activity (Ramakrishnan and Panachakel, 2021).

Sorkhabi (2014) used continuous wavelet transform (CWT) algorithm to divide EEG signals into 5 frequency bands. The CWT algorithm extracted variable-sized window features and obtained high-frequency information in shorter regions. Based on this, the researchers detected the activity of EEG signals within a 1-second time window. The subjects' self-rating values in terms of valence dimension and arousal degree were found to have a stable correlation with their EEG power and power spectrum entropy, and the high frequency band features had a more accurate classification effect than the low frequency band features. Atkinson and Campos (2016) proposed an emotion classification approach based on the combination of minimum redundancy-maximum correlation feature selection and kernel classifier. The significant benefit of this approach is that it incorporated the feature selection task of EEG signals into the classification task, identifying a wider range of emotion recognition approaches while using a multi-label classifier. Zhang and Lee, 2009 proposed an emotion understanding system. The system used the asymmetry of the prefrontal lobe of the brain as a feature and uses support vector machine (SVM) as a classifier, which can distinguish two emotional states with a recognition rate of about 73%. Zhuang et al. (2017) used the empirical mode decomposition (EMD) approach to automatically decompose the EEG signal into multiple intrinsic mode functions (IMFs), and achieved a recognition result of 70.41%. Mert and Akan (2018) used the multivariate extended version of the EMD approach to extract the multi-channel IMFs of EEG signal.

Neurobiological studies have shown that the human nervous system adopts a sparse representation strategy to receive and process external stimuli, and only needs to activate a small number of neurons in the cerebral cortex to complete the reception of information (Beyeler et al., 2019). Dictionary learning is a machine learning method based on this idea. In dictionary learning, data samples can be sparsely approximated by linear combinations of basis signals in the dictionary. The core idea is to learn an optimal dictionary under the certain constraint, and obtain the optimal sparse representation of the sample on the learned dictionary (Zhang et al., 2018). For EEG signals, we can also use sparse representation to avoid a lot of redundant information. Sheykhivand et al. (2020) took raw EEG signals directly as the model input and used a dictionary-learning-based sparse representation classifier. The classifier showed good performance without involving feature extraction and selection. Gu et al. (2021) mapped EEG signals in different frequency bands to subspaces, and learned a shared dictionary of multiple frequency bands, which can learn more discriminative knowledge hidden frequency bands. On the basis of this research, Zhu et al. (2022) proposed a new multi-band dictionary learning-based EEG emotion analysis model. They divided the projection matrix of each frequency band into two parts: common component and private component. The common component was used to mine the shared knowledge of different frequency bands, and the private component was used to mine the unique knowledge of each frequency band. Simultaneously, the shared dictionary on the multi-band signal in the subspace established the connection between multi-bands. Barthélemy et al. (2013) proposed a dictionary learning that considers both inter-channels links and shift-invariance, which improved the representation ability and flexibility of the dictionary. Although traditional EEG-based emotion classification approaches have been effectively verified and widely used, they are all based on subject-independent general models and do not consider individual differences. Studies have shown that subjects of different genders, ages, races, and health conditions have great differences in emotional expression, and sometimes this difference is even greater than the change in emotion (Chaplin, 2015). Therefore, the generalization performance of traditional emotion classification model is poor.

Traditional emotion classification model assumes that EEG signals from different subjects (training and test data) have the same feature space distribution. Transfer learning can relax this restriction. In target task modeling, transfer learning can use knowledge from other domain (source domain, SD) to help target domain (TD) training and modeling (Lin and Tzyy-Ping, 2017). For example, knowledge or patterns learned from other subjects can be applied to a new subject through transfer learning. By mining the information shared between different subjects, a model that adapts to the target subject's data distribution is finally constructed. In this study, a transfer discriminative dictionary pair learning approach (TDDPL) is developed for across-subject EEG emotion classification. The core idea of TDDPL is to find the domain-invariant subspace through the projection matrix, and learn the shared dictionary pair based on the paired dictionary learning framework in the subspace. By leveraging SD discriminative information, a shared synthesis dictionary and analysis dictionary are used to learn more discriminative domain-invariant low-rank coding to improve the performance of TD model. Our experiments in SEED and SEED IV datasets show that TDDPL achieves very competitive accuracy with state-of-the-art transfer learning approaches.

Specifically, the contribution of this work is fourfold. (1) By adopting the maximum mean discrepancy (MMD) of low-rank encoding to mitigate the distribution difference, EEG data from different subjects are projected into the domain-invariant subspace, and a bridge is built between SD and TD through transfer dictionary pair learning approach. (2) By minimizing the reconstruction error and inter-class separation of each synthesis sub-dictionary, the reconstruction between each sub-dictionary and heterogeneous low-rank coding is approximately an empty set, which can improve the discriminative ability of synthesis dictionary. (3) By minimizing the reconstruction error and the inter-class separation of each analysis sub-dictionary, the learned low-rank encoding has good sparsity. (4) The discriminative classifier in the TD is trained on the classifier parameter, analysis dictionary and projection matrix, the classifier can be directly used for the classification of test data, avoiding additional time-consuming coding reconstruction.

The remainder of this paper is organized as follows. Section Background introduces the study background. Section Domain Adaptation Sparse Representation Classifier the proposed approach. Section Experiment reports the experimental results. Section Conclusion draws conclusions.

## Background

### Transfer Leaning in EEG Emotion Classification

There are two general classification models for EEG emotion classification, one is to build an emotion classification model that can be used for a long time, and the other is to build an emotion classification model that can be used across subjects. For the same subject, the EEG signal will change over time. Compared with the differences in EEG signals between the same subjects, the differences in EEG signals between different subjects are greater, resulting in poor model generalization performance across subjects (Wan et al., 2021). In addition, in the research of emotion classification, building a general model often needs to acquire a large amount of subject data, which is often difficult to achieve. Therefore, the above two problems are how to mine the shared useful information in the EEG data with large differences, in which how to construct a general model across subject is more challenging.

Transfer learning is a machine learning strategy that uses existing knowledge to solve problems in different but related domains. The essence of transfer learning is the transfer and reuse of knowledge, which is, extracting useful knowledge from one or more SDs to assist in tasks in TD (Li et al., 2020). For example, Zanini et al. (2018) proposed a transfer learning model using Riemannian geometry to handle across-session and across-subject classification tasks in brain-computer interface (BCI). Using affine transformations of the spatial covariance matrices of data from each session or subject, the authors tackled EEG-based BCI across-subject classification problems, and then calibrated the classifier using data from previous sessions or subject data. Lan et al. (2018) combined domain adaptation approaches such as maximum independent domain adaptation, transfer component analysis, subspace alignment, and information theoretical learning with non-transfer learning approaches. The experimental results showed that the models using domain adaptation techniques outperform the non-transfer learning approaches. Zhang and Wu (2020) proposed a manifold embedded knowledge transfer learning model. This model aligned the covariance matrices of EEG data on a Riemannian manifold, and then performed domain adaptation by minimizing the difference in the joint probability distribution, while preserving the geometry of the original EEG data. Morioka et al. (2015) proposed a dictionary learning approach with strong generalization ability. To be suitable for emotion analysis of multiple individuals, the approach took the EEG signals of the target individual as the calibration data. Because EEG signals are weak and easily affected by noise, transfer learning for EEG emotion classification is still a very challenging research.

### EEG Emotion Datasets Used in This Study

The datasets used in this paper are experimented on two public emotion datasets SEED (Zheng and Lu, 2015) and SEED-IV (Zheng et al., 2019). The SEED dataset employed six emotion-labeled movie clips to elicit three emotions in subjects: positive, neutral, and negative. The subject's EEG data was recorded while inducing the subject's emotion, and the emotional label represented by the movie was the subject's EEG label. The mean age of the 15 Chinese subjects participating in the experiment was 23.27. Each subject performed a total of 3 trials, and each trial consisted of 15 trials. Each trial consisted of 5 s prompts, about 4 min of audio stimulation, 45s of self-assessment and 15 s of rest. Similarly, the SEED-IV dataset employed emotion-labeled movie clips to elicit four emotions in subjects: happy, sad, fearful, and neutral. The experiment recorded the subjects' EEG data at the same time, and the emotional label represented by the movie is the EEG label. The 15 healthy subjects who participated in the experiment were between the ages of 20 and 24. The experiment designed three different experiments for each subject, each experiment contained 24 trials (six trials for each emotion), and each experiment used a completely different movie clip. The specification comparison between the SEED and SEED-IV datasets is shown in Table 1.

**Table 1:** Specification comparison between the SEED and SEED-IV datasets.

	**SEED dataset**	**SEED-IV dataset**
Number of leads	62	62
Original sampling rate	1,000 Hz	1,000 Hz
Downsampling	200 Hz	200 Hz
Number of subjects	15	15
Emotional stimulation	Chinese movie clips	Chinese movie clips
Emotional types	Positive, neutral, negative	Positive, fear, neutral, negative
Number of sessions	3	3
Number of trials	15	24
Trial test length	about 4 min	about 2min

### Synthesis Dictionary and Analysis Dictionary Learning

Let $\text{Y}=\left[{\text{y}}_{1},...,{\text{y}}_{N}\right]\in {\text{R}}^{d\times N}$ be the data matrix. The core idea of synthesis dictionary learning looks for a dictionary **D** ∈ **R**^*d*×*r*^ that can express each sample, where *r* is number of dictionary atoms. Let **A** ∈ **R**^*r*×*N*^ be the coding coefficient matrix obtained by dictionary **D**. Synthesis dictionary learning model (Jiang et al., 2013) can be formulated by,


(1) $$\begin{array}{l} \underset{\text{D},\text{A}}{\min}\;{\left\|\text{Y}-\text{D}\text{A}\right\|}_{F}^{2}+{\left\|\text{A}\right\|}_{p}+f_{1}\left(\text{Y},\text{D},\text{A}\right), \end{array}$$

where ‖·‖_*p*_ is usually 0 or 1 norm. ${\left\|\text{Y}-\text{D}\text{A}\right\|}_{F}^{2}$ represents the reconstruction error. *f*_1_(·) can be some constraint terms on dictionary atoms, training samples and coding coefficients, such as low-rank constraint, label consistency constraint, locality constraint, structured sparsity constraint, and Fisher discriminative constraint (Wang et al., 2017). Synthesis dictionary learns a comprehensive dictionary by solving a reconstruction error minimization problem.

Analysis dictionary learning provides an intuitive explanation (Du et al., 2021). It directly acts dictionary on feature samples into sparse coding space, which is similar to the feature transformation. Analysis dictionary learning model can be formulated by


(2) $$\begin{array}{l} \underset{\text{P},\text{A}}{\min}\;{\left\|\text{P}\text{Y}-\text{A}\right\|}_{F}^{2}+{\left\|\text{A}\right\|}_{p}+f_{2}\left(\text{Y},\text{P},\text{A}\right), \end{array}$$

where **P** ∈ **R**^*d*×*r*^ is the learned synthesis dictionary. *f*_2_(·) can be some constraint terms to obtain the stable solution of **P** and **A**.

## Domain Adaptation Sparse Representation Classifier

### The Objective Function of TDDPL

Suppose the training set **Y** consists of *n* SD sets **Y**_*si*_(1 ≤ *i* ≤ *n*) and a TD set **Y**_*t*_,$\text{Y}=\left[{\text{Y}}_{s1},{\text{Y}}_{s2},...,{\text{Y}}_{sn},{\text{Y}}_{t}\right]\in {\text{R}}^{d\times N}$, where ${\text{Y}}_{si}\in {\text{R}}^{d\times N_{si}}$ and ${\text{Y}}_{t}\in {\text{R}}^{d\times N_{t}}$ composing of *k* classes of training samples, $N=\overset{n}{\underset{i=1}{\sum }}N_{si}+N_{t}$.

#### Synthesis Sub-dictionary Discriminative Term

The synthetic dictionary learning model learns a synthetic dictionary **D** to sparsely represent samples with a linear combination of a small number of dictionary atoms. We use the projection matrix to project the data of each domain into the subspace, and use the projected low-dimensional samples to learn a shared synthetic dictionary. Suppose the projection matrices corresponding to the *k*th class sample on the **Y**_*si*_ and **Y**_*t*_ are $\Omega_{si,k}\in {\text{R}}^{d\times m}$ and $\Omega_{t,k}\in {\text{R}}^{d\times m}$, respectively. *m* is the dimension of the projected subspace. $\Omega_{si,k}^{T}{\text{Y}}_{si,k}$ and $\Omega_{t,k}^{T}{\text{Y}}_{t}$ represent the *k*th class projecting samples belonging to SD and TD, respectively. The synthesis sub-dictionary discriminative term minimizes the reconstruction error term of each synthesis sub-dictionary, while minimizing the inter-class separation term. The designed synthesis sub-dictionary discriminative term is written as,


(3) $$\begin{array}{l} \underset{\Omega ,\text{D},\text{A}}{\min}\overset{K}{\underset{k=1}{\sum }}(\overset{n}{\underset{i=1}{\sum }}({\left\|\Omega_{si,k}^{T}{\text{Y}}_{si,k}-{\text{D}}_{k}{\text{A}}_{si,k}\right\|}_{F}^{2}+{\left\|{\text{D}}_{k}{\hat{\text{A}}}_{si,k}\right\|}_{F}^{2})\\ \;+{\left\|\Omega_{t,k}^{T}{\text{Y}}_{t,k}-{\text{D}}_{k}{\text{A}}_{t,k}\right\|}_{F}^{2}+{\left\|{\text{D}}_{k}{\hat{\text{A}}}_{t,k}\right\|}_{F}^{2}) \end{array}$$

where **A**_*si,k*_(**A**_*t,k*_) is the coding coefficients of **Y**_*si,k*_(**Y**_*t,k*_), and the ${\hat{\text{A}}}_{si,k}$(${\hat{\text{A}}}_{t,k}$) is the complement set of **A**_*si,k*_(**A**_*t,k*_), ${\hat{\text{A}}}_{si,k}⋃{\text{A}}_{si,k}=\text{A}$,${\hat{\text{A}}}_{si,k}⋂{\text{A}}_{si,k}=\emptyset$ (${\hat{\text{A}}}_{t,k}⋃{\text{A}}_{t,k}=\text{A}$,${\hat{\text{A}}}_{t,k}⋂{\text{A}}_{t,k}=\emptyset$). ${\text{D}}_{k}\in {\text{R}}^{m\times r_{k}}$ is the *k*th class sub-dictionary, where *r*_*k*_ is the number of sub-dictionary atoms.

The first item of Eq. (3) is to ensure the discriminative ability of the synthesis sub-dictionary, and each synthesis sub-dictionary **D**_*k*_ can represent the data well. The reconstruction structure of second item of Eq. (3) is approximately zero to achieve the separation between sub-dictionary classes.

Let $\Omega_{k}^{T}=\left[\Omega_{s1,k}^{T},...,\Omega_{sn,k}^{T},\Omega_{t,k}^{T}\right]$, [[Mathtype-mtef1-eqn-42.mtf]], ${\text{A}}_{k}=\left[{\text{A}}_{s1,k},...,{\text{A}}_{sn,k},{\text{A}}_{t,k}\right]$, Eq. (3) can be combined into the following form,


(4) $$\begin{array}{l} \underset{\Omega ,\text{D},\text{A}}{\min}\overset{K}{\underset{k=1}{\sum }}\left({\left\|\Omega_{k}^{T}{\text{Y}}_{k}-{\text{D}}_{k}{\text{A}}_{k}\right\|}_{F}^{2}+{\left\|{\text{D}}_{k}{\hat{\text{A}}}_{k}\right\|}_{F}^{2}\right) \end{array}$$

#### Analysis Sub-dictionary Discriminative Term

For the ${\text{Y}}_{si,k}$,${\text{P}}_{k}\Omega_{si,k}^{T}{\text{Y}}_{si,k}$ represents the coding coefficients of $\Omega_{si,k}^{T}{\text{Y}}_{si,k}$ on the analysis sub-dictionary ${\text{P}}_{k}\in {\text{R}}^{m\times r_{k}}$. To achieve the discriminative ability of each **P**_*k*_, the *k*-th low-dimensional projection data is projected into a non-zero encoding space, i.e., ${\text{P}}_{k}\Omega_{si,k}^{T}{\text{Y}}_{si,k}\approx {\text{A}}_{si,k}$. Simultaneously, the *j*-th low-dimensional projection data is projected into approximate zero encoding space, i.e., ${\text{P}}_{k}\Omega_{sj,k}^{T}{\text{Y}}_{si,j}\approx 0,\forall k\neq j$.

Implement this idea on all SDs and TD, the designed analysis sub-dictionary discriminative term is written as,


(5) $$\begin{array}{l} \underset{\Omega ,\text{D},\text{A}}{\min}\overset{K}{\underset{k=1}{\sum }}(\overset{n}{\underset{i=1}{\sum }}\left({\left\|{\text{P}}_{k}\Omega_{si,k}^{T}{\text{Y}}_{si,k}-{\text{A}}_{si,k}\right\|}_{F}^{2}+{\left\|{\text{P}}_{k}\Omega_{si,k}^{T}{\text{Y}}_{si,k}{\hat{\text{A}}}_{si,k}\right\|}_{F}^{2}\right)\\ \;+{\left\|{\text{P}}_{k}\Omega_{t,k}^{T}{\text{Y}}_{t,k}-{\text{A}}_{t,k}{\|}_{F}^{2}+\|{\text{P}}_{k}\Omega_{si,k}^{T}{\text{Y}}_{si,k}{\hat{\text{A}}}_{t,k}\right\|}_{F}^{2}) \end{array}$$

Let ${\text{P}}_{k}=\left[{\text{P}}_{s1,k},...,{\text{P}}_{sn,k},{\text{P}}_{t,k}\right]$, Eq. (5) can be combined into the following form,


(6) $$\begin{array}{l} \underset{\Omega ,\text{D},\text{A}}{\min}\overset{K}{\underset{k=1}{\sum }}\left({\left\|{\text{P}}_{k}\Omega_{k}^{T}{\text{Y}}_{k}-{\text{A}}_{k}\right\|}_{F}^{2}+{\left\|{\text{P}}_{k}\Omega_{k}^{T}{\text{Y}}_{k}{\hat{\text{A}}}_{k}\right\|}_{F}^{2}\right) \end{array}$$

#### Domain Adaptation Term

In the projected subspace, the difference of data distribution between SD and TD is measured on low-rank coding using MMD strategy. The designed domain adaptation term is written as,


(7) $$\begin{array}{l} \underset{\text{A}}{\min}\;\overset{K}{\underset{k=1}{\sum }}{\left\|\frac{1}{N_{k}^{s}}\overset{N_{k}^{s}}{\underset{i=1}{\sum }}{\text{a}}_{i}^{s}-\frac{1}{N_{k}^{t}}\overset{N_{k}^{t}}{\underset{i=1}{\sum }}{\text{a}}_{j}^{t}\right\|}_{2}^{2} \end{array}$$

Let


$$\begin{array}{l} Q_{k}^{ij}=\{\begin{array}{cc} \frac{1}{N_{k}^{s}N_{k}^{s}}, & if\;{\text{y}}_{i},{\text{y}}_{j}\;is\;from\;SD\\ \frac{1}{N_{k}^{t}N_{k}^{t}}, & if\;{\text{y}}_{i},{\text{y}}_{j}\;is\;from\;TD\\ \frac{-1}{N_{k}^{s}N_{k}^{t}}, & if\;{\text{y}}_{i}\left({\text{y}}_{j}\right)\;is\;from\;SD,\;and\;{\text{y}}_{j}\left({\text{y}}_{i}\right)\;is\;from\;TD\\ 0, & \;otherwise \end{array} \end{array}$$

Eq. (7) can be written in the matrix form,


(8) $$\begin{array}{l} \underset{\text{A}}{\min}\;\overset{K}{\underset{k=1}{\sum }}\overset{N_{k}^{s}}{\underset{i=1}{\sum }}\overset{N_{k}^{t}}{\underset{j=1}{\sum }}{\text{a}}_{i}^{T}{\text{a}}_{j}{\text{Q}}_{ij}^{k}=\underset{\text{A}}{\min}\;\overset{K}{\underset{k=1}{\sum }}tr\left({\text{A}}_{k}{\text{Q}}_{k}{\text{A}}_{k}^{T}\right) \end{array}$$

where low-rank matrix $\text{A}=\left[{\text{A}}_{s1},...,{\text{A}}_{sm},{\text{A}}_{t}\right]$.

#### Explicit Rank Minimization Term

Imposing low-rank constraints on the coding coefficients can significantly reduce the adverse effects of noise in the samples. A low-rank constraint is imposed on each analysis in this study. To improve computing efficiency of low-rank computation, following (Ding and Fu, 2019), the low-rank coding matrix is expressed as the product of two matrices, **A** ≈ **ΛΘ**, where **Λ** ∈ **R**^*m*×*K*^ and **Θ** ∈ **R**^*K*×*N*^. The designed explicit rank minimization term is written as,


(9) $$\begin{array}{l} rank\left(\text{A}\right)=\overset{K}{\underset{k=1}{\sum }}{\left\|{\text{A}}_{k}-\Lambda_{k}\Theta_{k}\right\|}_{F}^{2} \end{array}$$

#### Discriminative Classifier Term

A linear classifier based on low rank coding is embedded in the TDDPL approach. Let *W*_*k*_ and **H**_*k*_ be the classifier parameter and class label of *k*-th class data, respectively. For **W**_*k*_ and **H**_*k*_ with the same class, the ideal classification result is **W**_*k*_**P**_*k*_**X**_*k*_ ≈ **H**_*k*_. According to this idea, the designed discriminative classifier term is written as,


(10) $$\begin{array}{l} \underset{\text{P}\text{,}\text{W},\Omega }{\text{min}}\overset{K}{\underset{k=1}{\sum }}{\left\|{\text{H}}_{k}-{\text{W}}_{k}{\text{P}}_{k}\Omega_{k}^{T}{\text{Y}}_{k}\right\|}_{F}^{2} \end{array}$$

Considering Eqs. (4), (6), (8)-(10), the objective function of TDDPL can be written as,


(11) $$\begin{array}{l} \underset{\Omega ,\text{D},\text{P},\text{A},\Lambda ,\Theta ,\text{W}}{\min}\overset{K}{\underset{k=1}{\sum }}({\left\|\Omega_{k}^{T}{\text{Y}}_{k}-{\text{D}}_{k}{\text{A}}_{k}{\|}_{F}^{2}+\|{\text{D}}_{k}{\hat{\text{A}}}_{k}\right\|}_{F}^{2}\\ +\lambda_{1}({\left\|{\text{P}}_{k}\Omega_{k}^{T}{\text{Y}}_{k}-{\text{A}}_{k}\right\|}_{F}^{2}+{\left\|{\text{P}}_{k}\Omega_{k}^{T}{\text{Y}}_{k}{\hat{\text{A}}}_{k}\right\|}_{F}^{2})+\lambda_{2}tr\left({\text{A}}_{k}{\text{Q}}_{k}{\text{A}}_{k}^{T}\right)\\ +\lambda_{3}{\left\|{\text{A}}_{k}-\Lambda_{k}\Theta_{k}\right\|}_{F}^{2}+\lambda_{4}{\left\|{\text{H}}_{k}-{\text{W}}_{k}{\text{P}}_{k}\Omega_{k}^{T}{\text{Y}}_{k}\right\|}_{F}^{2})\\ +\gamma {\left\|\text{W}\right\|}_{F}^{2}. \end{array}$$

Obviously, we can obtain the projection matrix **Ω**, synthesis dictionary **D**, analysis dictionary **P**, low-rank coding **A** (matrices **Λ** and **Θ**) by solving the optimization Eq. (11). The projection matrix **Ω** projects the SD and TD samples into a low-dimensional subspace. According to the MMD strategy, the data distribution differences between different domains are as small as possible. The synthesis dictionary **D** can better reconstruct the projection sample Ω^*T*^**Y**. The multiplication of the analysis dictionary **P** and the projection sample Ω^*T*^**Y**, i.e., **P**Ω^*T*^**Y** can obtain an approximate block diagonal coding coefficient matrix with strong discriminative ability. The shared dictionary pair **P** and **D** become the bridges between SD and TD. The discriminative knowledge of SD is transferred to TD space to build a discriminative classifier.

### Optimization

Eq. (11) is a non-convex optimization problem. However, it is a convex optimization problem when only one variable is optimized while fixing the other variables. Therefore, the optimization problem Eq. (11) is split into several sub-optimization problems here.

Let $\Omega_{k}^{T}{\text{Y}}_{k}={\text{B}}_{k}$,${\text{P}}_{k}\Omega_{k}^{T}{\text{Y}}_{k}={\text{C}}_{k}$, we have **P**_*k*_**B**_*k*_ = **C**_*k*_. Eq. (11) can be re-written as,


(12) $$\begin{array}{l} \underset{\begin{array}{c} \Omega ,\text{D},\text{P},\text{A},\\ \Lambda ,\Theta \text{,}\text{B}\text{,}\text{C},\text{W} \end{array}}{\min}\overset{K}{\underset{k=1}{\sum }}({\left\|{\text{B}}_{k}-{\text{D}}_{k}{\text{A}}_{k}{\|}_{F}^{2}+\|{\text{D}}_{k}{\hat{\text{A}}}_{k}\right\|}_{F}^{2}+\lambda_{1}({\left\|{\text{C}}_{k}-{\text{A}}_{k}\right\|}_{F}^{2}\\ \;+{\left\|{\text{C}}_{k}{\hat{\text{A}}}_{k}\right\|}_{F}^{2})+\lambda_{2}tr({\text{A}}_{k}{\text{Q}}_{k}{\text{A}}_{k}^{T})+\lambda_{3}{\left\|{\text{A}}_{k}-\Lambda_{k}\Theta_{k}\right\|}_{F}^{2}\\ \;+\lambda_{4}{\left\|{\text{H}}_{k}-{\text{W}}_{k}{\text{C}}_{k}\right\|}_{F}^{2}+\lambda_{5}({\left\|{\text{B}}_{k}-\Omega_{k}^{T}{\text{Y}}_{k}\right\|}_{F}^{2}\\ \;+{\left\|{\text{C}}_{k}-{\text{P}}_{k}{\text{B}}_{k}\right\|}_{F}^{2}))+\gamma {\left\|\text{W}\right\|}_{F}^{2},\\ s.t.\;{\left\|{\text{d}}_{i}\right\|}^{2}=1,\;\forall i \end{array}$$

1) Update **A**, while fixing **Ω**, **D**, **P**, **Λ**, **Θ**, **B**, **C**, **W**.

To remove terms which are irrelevant to **A**, we have,


(13) $$\begin{array}{l} \left[\;\text{A}\right]=\overset{K}{\underset{k=1}{\sum }}\left({\left\|{\text{B}}_{k}-{\text{D}}_{k}{\text{A}}_{k}\right\|}_{F}^{2}+\lambda_{1}{\left\|{\text{C}}_{k}-{\text{A}}_{k}\right\|}_{F}^{2}+\lambda_{2}tr({\text{A}}_{k}{\text{Q}}_{k}{\text{A}}_{k}^{T}\right)\\ \;+\lambda_{3}{\left\|{\text{A}}_{k}-\Lambda_{k}\Theta_{k}\right\|}_{F}^{2}), \end{array}$$

By setting the derivative of Eq. (13) to zero, we obtain the solution of **A**_*k*_,


(14) $$\begin{array}{l} {\text{A}}_{k}={\left({\text{D}}_{k}^{T}{\text{D}}_{k}+(\lambda_{1}+\lambda_{3})\text{I}+\lambda_{2}{\text{Q}}_{k}\right)}^{-1}({\text{D}}_{k}^{T}{\text{B}}_{k}+\lambda_{1}{\text{C}}_{k}+\lambda_{3}\Lambda_{k}\Theta_{k}) \end{array}$$

2) Update **P**, while fixing **Ω**, **D**, **A**, **Λ**, **Θ**, **B**, **C**, **W**.

To remove terms which are irrelevant to **P**, we have,


(15) $$\begin{array}{l} \left[\text{P}\right]=\overset{K}{\underset{k=1}{\sum }}\left({\left\|{\text{C}}_{k}-{\text{P}}_{k}{\text{B}}_{k}\right\|}_{F}^{2}\right), \end{array}$$

By setting the derivative of Eq. (15) to zero, we obtain the solution of **P**,


(16) $$\begin{array}{l} {\text{P}}_{k}={\text{C}}_{k}{\text{B}}_{k}^{T}{\text{(}{\text{B}\text{B}}_{k}^{T}+\theta \text{I})}^{-1} \end{array}$$

where θ**I** is to prevent singular solution in matrix inversion.

3) Update **W**, while fixing **Ω**, **D**, **P**, **A**, **Λ**, **Θ**, **B**, **C**.

To remove terms which are irrelevant to **W**, we have,


(17) $$\begin{array}{l} \;\left[\;\text{W}\;\right]=\overset{K}{\underset{k=1}{\sum }}\left(\lambda_{4}{\left\|{\text{H}}_{k}-{\text{W}}_{k}{\text{C}}_{k}\right\|}_{F}^{2}\right)+\gamma {\left\|\text{W}\right\|}_{F}^{2} \end{array}$$

By setting the derivative of Eq. (17) to zero, we obtain the solution of ${\text{W}}_{k}$,


(18) $$\begin{array}{l} {\text{W}}_{k}=\left(\lambda_{4}{\text{H}}_{k}{\text{C}}_{k}^{T}\right){\left(\lambda_{4}{\text{C}}_{k}{\text{C}}_{k}^{T}+\gamma \text{I}\right)}^{-1} \end{array}$$

4) Update **D**, while fixing **Ω**, **P**, **A**, **Λ**, **Θ**, **B**, **C**, **W**.

To remove terms which are irrelevant to **D**, we have,


(19) $$\begin{array}{l} \begin{array}{c} \;\left[\;\text{D}\;\right]=\overset{K}{\underset{k=1}{\sum }}\left({\left\|{\text{B}}_{k}-{\text{D}}_{k}{\text{A}}_{k}\right\|}_{F}^{2}+{\left\|{\text{D}}_{k}{\hat{\text{A}}}_{k}\right\|}_{F}^{2}\right),\\ s.t.\;{\left\|{\text{d}}_{i}\right\|}_{2}^{2}\leq 1,\;\forall i \end{array} \end{array}$$

Eq. (19) can be solved via the dual from, then we obtain,


(20) $$\begin{array}{l} \;\left[\;{\text{D}}_{k}\;\right]={\left\|{\text{B}}_{k}-{\text{D}}_{k}{\text{A}}_{k}\right\|}_{F}^{2}+{\left\|{\text{D}}_{k}{\hat{\text{A}}}_{k}\right\|}_{F}^{2}+\overset{n}{\underset{i=1}{\sum }}\sigma_{k,i}\left({\left\|{\text{d}}_{i}\right\|}^{2}-1\right) \end{array}$$

where σ_*k,i*_ is the Lagrange multiplier of the *i* th equality constraint.

Let $\Gamma_{k}\in {\text{R}}^{m\times m}$[[Mathtype-mtef1-eqn-105.mtf]], where ${\left(\Gamma_{k}\right)}_{i,i}\in {\text{R}}^{m\times m}$,(_**Γ**_*k*_)*i, i*_ = σ_*i*_, we obtain,


(21) $$\begin{array}{l} L({\text{D}}_{k}\text{,}{\text{d}}_{i})={\left\|{\text{B}}_{k}-{\text{D}}_{k}{\text{A}}_{k}\right\|}_{F}^{2}+{\left\|{\text{D}}_{k}{\hat{\text{A}}}_{k}\right\|}_{F}^{2}\text{+}tr\text{(}{\text{D}}_{k}^{T}{\text{D}}_{k}\Gamma_{k}\text{)}-tr\left(\Gamma_{k}\right) \end{array}$$

By setting the derivative of Eq. (21) to zero, we obtain the solution of ${\text{D}}_{k}$,


(22) $$\begin{array}{l} {\text{D}}_{k}={\text{B}}_{k}{\text{A}}_{k}^{T}{\text{(}{\text{A}}_{k}{\text{A}}_{k}^{T}\text{+}{\hat{\text{A}}}_{k}{\text{(}{\hat{\text{A}}}_{k}\text{)}}^{T}\text{+}\Gamma_{k}\text{)}}^{-1} \end{array}$$

5) Update **Λ**and **Θ**, while fixing **Ω**, **P**, **A**, **D**, **B**, **C**, **W**.

To remove terms which are irrelevant to **Λ** and **Θ**, we have,


(23) $$\begin{array}{l} \left[\Lambda_{k},\Theta_{k}\;\right]=\overset{K}{\underset{k=1}{\sum }}{\left\|{\text{A}}_{k}-\Lambda_{k}\Theta_{k}\right\|}_{F}^{2} \end{array}$$

By setting the derivative of Eq. (23) to zero, we obtain the solution of **Λ**_*k*_ and **Θ**_*k*_,


(24) $$\begin{array}{l} \Lambda_{k}={\text{A}}_{k}\Theta_{k}^{T}{\text{(}\Theta_{k}\Theta_{k}^{T}\text{)}}^{†} \end{array}$$

(25) $$\begin{array}{l} \Theta_{k}\text{=(}\Lambda_{k}\Lambda_{k}^{T}{\text{)}}^{†}\Lambda_{k}^{T}\Lambda_{k} \end{array}$$

where (▪)^†^is the Moore–Penrose pseudoinverse.

6) Update **Ω**, while fixing **P**, **A**, **Λ**, **Θ**, **D**, **B**, **C**, **W**.

To remove terms which are irrelevant to **Ω**, we have,


(26) $$\begin{array}{l} \left[\Omega \right]=\overset{K}{\underset{k=1}{\sum }}{\left\|{\text{B}}_{k}-\Omega_{k}^{T}{\text{Y}}_{k}\right\|}_{F}^{2} \end{array}$$

To set the derivative of Eq. (26) with respect to **Ω** to zero, we have,


(27) $$\begin{array}{l} \Omega_{k}={\text{B}}_{k}{\text{Y}}_{k}^{T}{\left({\text{Y}}_{k}{\text{Y}}_{k}^{T}+\theta \text{I}\right)}^{-1} \end{array}$$

where θ**I** is to prevent singular solution in matrix inversion.

7) Update and **C**, while fixing.

To remove terms which are irrelevant to **C**_*k*_, we have,


(28) $$\begin{array}{l} {}[{\text{C}}_{k}]=\sum_{k=1}^{K}(\lambda_{1}({\left\|{\text{C}}_{k}-{\text{A}}_{k}\right\|}_{F}^{2}+{\left\|{\text{C}}_{k}{\hat{A}}_{k}\right\|}_{F}^{2})+\lambda_{1}\|{\text{H}}_{k}\\ \;{-{\text{W}}_{k}{\text{C}}_{k}\|}_{F}^{2}+\lambda_{5}{\left\|{\text{C}}_{k}-{\text{P}}_{k}{\text{B}}_{k}\right\|}_{F}^{2}), \end{array}$$

We use sub-gradient descent method, and compute the gradient of Eq.(28) with respect to **C**_*k*_,


(29) $$\begin{array}{l} \frac{\partial \left[{\text{C}}_{k}\right]}{\partial {\text{C}}_{k}}=\lambda_{1}\left({\text{C}}_{k}-{\text{A}}_{k}+{\text{C}}_{k}{\hat{\text{A}}}_{k}{\hat{\text{A}}}_{k}^{T}\right)+\lambda_{4}\left({\text{W}}_{k}^{T}{\text{W}}_{k}{\text{C}}_{k}-{\text{W}}_{k}^{T}{\text{H}}_{k}\right)\\ \;+\lambda_{5}\left({\text{C}}_{k}-{\text{P}}_{k}{\text{B}}_{k}\right) \end{array}$$

**C**_*k*_ can be updated by descent method with learning rate α,


(30) $$\begin{array}{l} {\text{C}}_{k}={\text{C}}_{k}-\alpha \frac{\partial \left[{\text{C}}_{k}\right]}{\partial {\text{C}}_{k}} \end{array}$$

To remove terms which are irrelevant to **B**_*k*_, we have,


(31) $$\begin{array}{l} {}[B_{k}]=\sum_{k=1}^{K}({\left\|B_{k}-D_{k}A_{k}\right\|}_{F}^{2}+\lambda_{5}({\left\|B_{k}-\Omega_{k}^{T}\right\|}_{F}^{2}\\ \;+{\left\|C_{k}-P_{k}B_{k}\right\|}_{F}^{2})), \end{array}$$

By setting the derivative of Eq. (31) to zero, we obtain the solution of **B**_*k*_,


(32) $$\begin{array}{l} {\text{B}}_{k}={\left(\left(1+\lambda_{5}\right)\text{I}+\lambda_{5}{\text{P}}_{k}^{T}{\text{P}}_{k}\right)}^{-1}\left({\text{D}}_{k}{\text{A}}_{k}+\lambda_{5}\Omega_{k}^{T}{\text{Y}}_{k}+\lambda_{5}{\text{P}}_{k}^{T}{\text{C}}_{k}\right) \end{array}$$

Algorithm 1 describes the proposed TDDPL approach.

**Algorithm 1:** Transfer discriminative dictionary pair learning approach.

Input: *n* SD training sets **Y**_*si*_(1 ≤ *i* ≤ *n*)and a TD set **Y**_*t*_.
Output: parameters {Ω,**P**, **A**, Λ, Θ, **D**, **B**, **C**, **W**}
Initialize: initialize **P**,**D** and **A** by dictionary pair learning (DPL) approach, Ω as the random matrix; initialize Λ = **I**, Θ = **I**, **B** = **I**, **C** = **I**, and **W** = **I**;
While not converge do
Fixing Ω, **D**, **P**, Λ, Θ, **B**, **C**, **W**, update **A** by solving Eq. (14);
Fixing Ω, **D**, **A**, Λ, Θ, **B**, **C**, **W**, update **P** by solving Eq. (16);
Fixing Ω, *D, P, A*, Λ, Θ, *B, C*, update **W** by solving Eq. (18);
Fixing Ω, **P**, **A**, Λ, Θ, **B**, **C**, **W**, update **D** by solving Eq. (22);
Fixing Ω, **P**, **A**, **D**, **B**, **C**, **W**, update Λand Θ by solving Eqs. (24)-(25);
Fixing **P**, **A**, Λ, Θ, **D**, **B**, **C**, **W**, update Ω by solving Eqs. (27);
Fixing **P**, **A**, Λ, Ω, Θ, **D**, **W**, update **C** and **B** by solving Eqs. (30)–(32);
end while

### Testing

After obtaining the classifier parameter **W**, projection matrix **Ω**, and analysis sub-dictionary **P**, we classify the test samples based on **W**, **Ω**, and **P**. For test sample **x**_*new*_, we use the following formulation to predict its class label,


(33) $$\begin{array}{l} l\left({\text{x}}_{new}\right)=\arg\max_{i\leq c}{\left(\text{W}\text{P}\Omega^{T}{\text{x}}_{new}\right)}_{i} \end{array}$$

where ${\left(\text{W}\text{P}\Omega^{T}{\text{x}}_{new}\right)}_{i}$ is the *i*-th element of $\text{W}\text{P}\Omega^{T}{\text{x}}_{new}$.

## Experiment

### Experimental Setting

For the SEED dataset, EEG features were extracted in non-overlapping 1s time windows for each segment of preprocessed EEG data. For the SEED-IV dataset, EEG features were extracted in non-overlapping 4 s time windows for each segment of preprocessed EEG data. The feature extraction approach used in this paper is the differential entropy (DE) feature (Li et al., 2019). In the experiment, DE features are calculated in 5 frequency bands for each channel. So the total dimension of the extracted features is 62 × 5=310 dimensions. We evaluate the TDDPL approach on two transfer learning strategies: one SD to one TD (o → o), and multiple SDs to one TD (m → o). For the o → o scenario, we choose one subject used as the TD and another subject used as the SD. If z is the number of subjects in the SEED and SEED IV datasets, there are z(z-1) different o → o tasks in total. In the SEED dataset, for the data in the TD, the 30 s data of each trial is randomly selected for training, and the rest of the data is used for testing. In the SEED-IV dataset, for the data in the TD, the 28 s data of each trial is randomly selected for training, and the rest of the data is used for testing. For the m → o scenario, one subject is selected as the TD and all the remaining subjects are used as the SD, so there are z different tasks in the m → o scenario. Due to the large number of training samples in the SD, for the SEED dataset and SEED-IV datasets, we select 1/10 of the SD data for training, and repeat this process 10 times, so that the randomly sampled training data can cover the entire training dataset. The selection of training set and test set of the TD is consistent with the o → o scenario.

We compare the TDDPL approach with the following approaches: 1) Traditional machine learning approaches: sparse representation-based classification (SRC) (Wright et al., 2009) and DPL approach (Ameri et al., 2016). 2) Transfer learning approaches: manifold embedded knowledge transfer (MEKT) (Zhang and Wu, 2020), transfer component analysis (TCA) (Pan et al., 2011), maximum independence domain adaptation (MIDA) (Yan et al., 2018), domain adaptation sparse representation classifier (DASRC) (Ni et al., 2021). For TCA and MEKT, the Gaussian kernel parameter and regularization parameters are set in the grid {2^−6^,..., 2^5^}. For SRC, DPL, DASRC, and TDDPL, the dimension of subspace and the number of atoms in each class are set in the grid {20, 30,..., 100} and {10, 15,..., 30}, respectively. All the algorithms are implemented in MATLAB.

### Experiments on the SEED Dataset

In this subsection, we compare the approaches on DE feature of the SEED dataset. Tables 2, 3 show the average accuracy of each approach for the o → o and m → o scenarios on the session 1, respectively. It can be seen that compared with other approaches, (1) the classification performances of the approaches using the transfer learning strategy outperform those of the approaches without the transfer learning strategy. It is demonstrated that transfer learning strategy can mitigate the impact of individual differences to a certain extent. Since the SRC and DPL classifiers simply combine SD and TD to build a model which may not be adapted to the test set data distribution, it is difficult to obtain ideal experimental results. It also indicates that transfer learning improves the approach's classification performance in across-subject EEG emotion analysis. (2) Regardless of the o → o or m → o scenarios, the TDDPL approach achieves the best or second best performance in Tables 2, 3. As seen the results in Table 2, the TDDPL approach improves by 1.70% compared with the best comparison approach. The TDDPL approach projects the EEG data of different subjects into the subspace to reduce the distribution difference between subjects. The shared analysis dictionary learned by the TDDPL produces discriminative low-rank coding, and the learned shared synthesis dictionary has good coding reconstruction ability. Therefore, the TDDPL approach performs well and is more stable in across-subject EEG emotion classification.

**Table 2:** Average classification accuracies on session 1 of SEED dataset in the o → o scenario.

**Subject**	**SRC**	**DPL**	**TCA**	**MIDA**	**MEKT**	**DASRC**	**TDDPL**
1	52.16	54.65	55.66	52.83	60.68	62.68	**68.04**
2	52.05	52.08	55.09	57.65	60.93	64.94	**66.64**
3	54.38	54.53	57.61	60.87	61.21	63.21	**66.16**
4	48.58	55.73	59.33	62.54	69.12	70.12	**70.30**
5	56.29	55.05	56.30	58.73	58.27	60.27	**63.13**
6	54.19	60.04	56.35	59.27	64.28	66.28	**68.65**
7	50.40	49.32	55.25	58.00	56.57	**58.57**	57.44
8	56.34	49.47	53.60	55.43	54.94	56.93	**58.63**
9	55.68	62.64	58.52	60.84	65.78	**67.78**	66.84
10	53.62	48.35	55.61	58.75	58.80	60.80	**63.21**
11	53.93	52.00	59.75	61.75	62.46	64.45	**66.51**
12	42.74	59.67	63.35	64.64	64.88	66.89	**69.52**
13	52.71	61.53	59.16	55.51	66.67	68.67	**70.79**
14	56.14	57.64	56.05	54.25	59.11	60.11	**60.21**
15	56.21	61.11	66.62	68.42	69.07	72.08	**73.12**
Average	53.03	55.59	57.88	59.30	62.18	64.25	**65.95**

*The bold values in **Tables** 2–5 mean the best values in comparison experiments*.

**Table 3:** Average classification accuracies on session 1 of SEED dataset in the m → o scenario.

**Subject**	**SRC**	**DPL**	**TCA**	**MIDA**	**MEKT**	**DASRC**	**TDDPL**
1	59.42	61.25	70.94	66.96	74.59	75.70	**81.83**
2	58.58	58.46	70.26	73.55	75.52	77.15	**81.34**
3	59.89	61.27	73.27	74.83	74.43	77.32	**80.29**
4	54.21	61.91	75.15	76.15	83.12	83.78	**84.48**
5	62.15	61.47	71.41	73.49	72.46	74.19	**77.26**
6	60.41	66.48	71.13	73.27	78.55	79.72	**82.32**
7	57.50	55.79	70.83	71.10	70.86	**72.71**	71.50
8	63.50	55.03	68.52	69.73	68.41	71.06	**73.04**
9	61.36	67.37	73.07	75.10	80.13	**81.25**	80.40
10	60.37	54.03	72.01	73.02	72.60	73.83	**76.41**
11	60.32	56.54	73.95	75.90	76.57	78.02	**80.26**
12	48.67	65.70	77.51	78.28	78.34	79.16	**82.51**
13	58.35	66.38	71.63	69.53	80.03	82.27	**83.67**
14	62.06	62.85	66.46	68.40	73.27	73.49	**74.45**
15	63.11	66.31	80.56	81.18	83.23	84.62	**85.81**
Average	59.33	61.39	72.45	73.37	76.14	77.62	**79.70**

**Figure 1:**
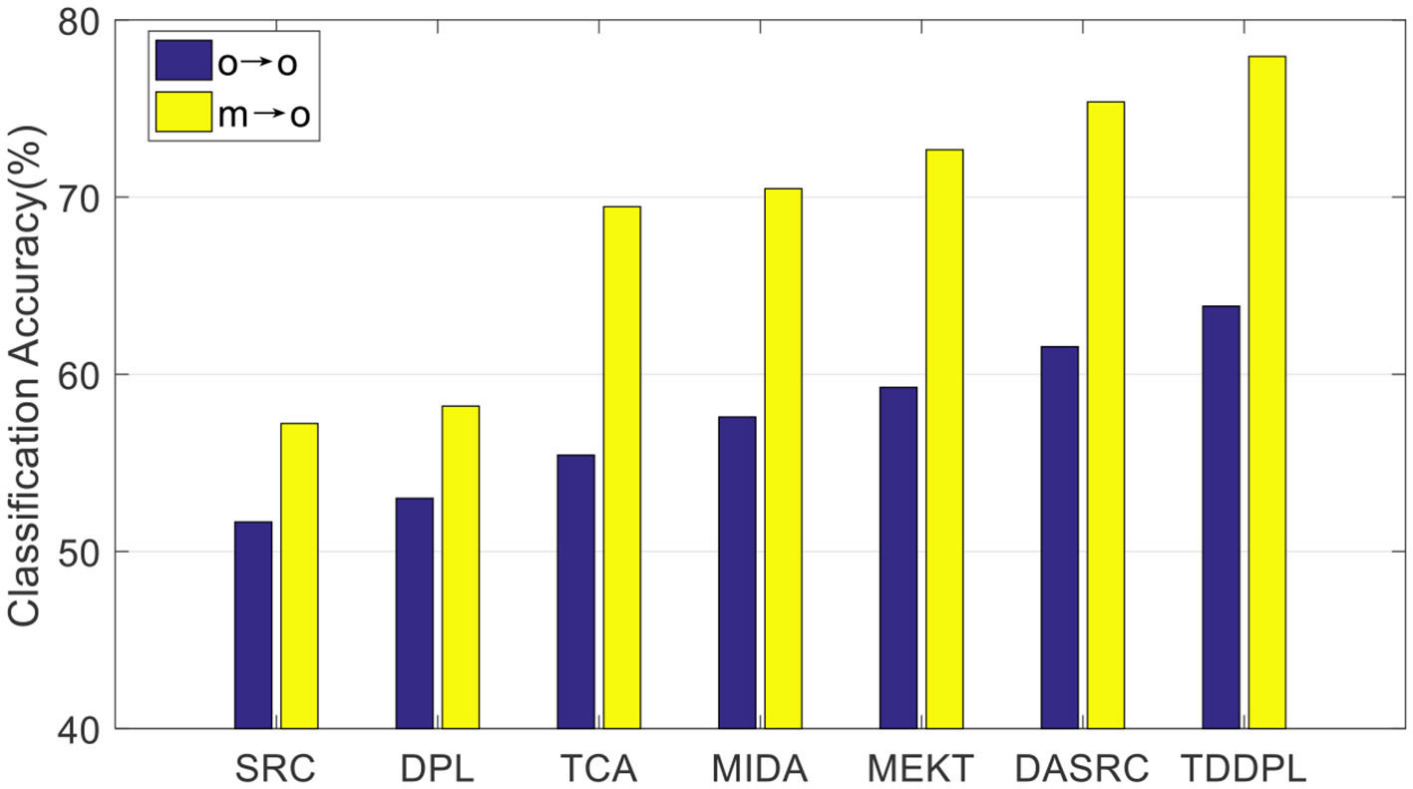
Average classification accuracies on session 2 of the SEED dataset.

**Figure 2:**
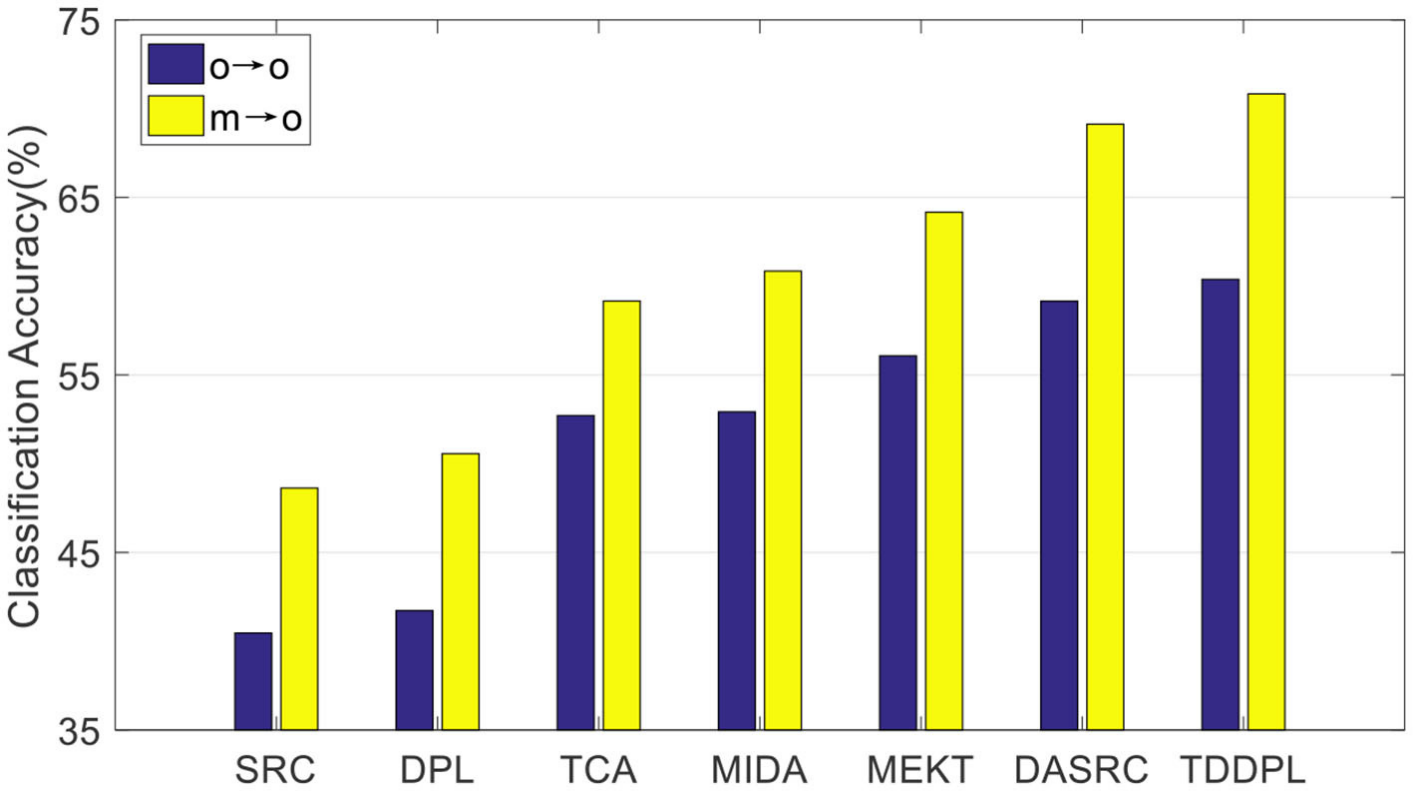
Average classification accuracies on session 3 of the SEED dataset.

To verify the performance of our approach on other commonly used EEG features, we compared the average accuracy of the TDDPL approach with other approaches on session 2 and session 3 of the SEED dataset. The average classification accuracy is shown in Figures 1, 2. As shown in Figures 1, 2, the TDDPL approach has the best average classification accuracy on session 2 and session 3, and the experimental results are significantly better than other comparison approaches. The TDDPL approach improves by 10.36% compared with the traditional DPL classification approach, and also improves by 1.70% compared with the best comparison approach in the o → o scenario. The results reveal that the TDDPL approach has excellent across-subject adaptability, and it can accurately and effectively realize EEG emotion classification.

### Experiments on the SEED IV Dataset

Next, we compare the approaches on the DE feature of the SEED IV dataset. Tables 4, 5 show the average classification accuracy of each approach for the o → o scenario and the m → o scenario on the session 1, respectively. As can be seen in Tables 4, 5, (1) Because the emotion classes of SEED IV dataset are more complex than the SEED dataset, the average accuracy of each approach is slightly lower than that of Tables 2, 3. (2) Similar to the results in Tables 2, 3, the transfer learning approaches (MIDA, MEKT, and DASRC) outperforms the non-transfer learning approaches (SRC and DPL), which indicates that simply mixing SD and TD into a training set is not suitable for EEG emotion recognition. (3) TDDPL achieved the best results on the vast majority of individuals. The label information of the SD data is transferred to TD through the shared dictionary pair **D** and **P**, thereby improving the performance of the task in TD. TDDPL also combines the advantages of a comprehensive dictionary approach and an analytical dictionary approach. Based on the learned analysis dictionary, projection matrix, and classifier parameters, the classifier learned in TD has good discriminative ability.

**Table 4:** Average classification accuracies on session 1 of SEED IV dataset in the o → o scenario.

**Subject**	**SRC**	**DPL**	**TCA**	**MIDA**	**MEKT**	**DASRC**	**TDDPL**
1	38.78	40.41	50.47	48.63	54.56	54.36	**54.82**
2	43.81	42.88	56.74	55.78	56.95	63.72	**65.86**
3	42.77	43.61	50.74	48.55	53.34	56.82	**56.84**
4	31.91	33.00	51.41	54.16	58.97	60.47	**66.11**
5	39.61	40.53	41.38	42.04	43.18	50.32	**50.75**
6	34.96	35.34	47.89	47.56	55.08	58.42	**61.78**
7	42.24	43.39	54.67	56.50	59.32	61.48	**61.74**
8	38.36	39.37	48.70	48.55	56.02	**59.59**	58.66
9	41.97	43.05	48.06	46.42	49.18	47.37	**47.66**
10	38.73	39.78	49.32	48.27	52.89	52.95	**53.22**
11	33.63	33.00	39.28	39.32	40.66	43.96	**44.34**
12	30.29	33.27	36.02	38.24	37.08	39.85	**40.71**
13	37.85	36.79	48.96	48.11	46.00	49.31	**55.39**
14	37.23	40.18	45.25	44.63	50.26	50.21	**50.62**
15	41.26	43.10	58.45	57.84	62.66	63.64	**64.66**
Average	38.23	39.18	48.49	48.31	51.74	54.16	**55.54**

**Table 5:** Average classification accuracies on session 1 of SEED IV dataset in the m → o scenario.

**Subject**	**SRC**	**DPL**	**TCA**	**MIDA**	**MEKT**	**DASRC**	**TDDPL**
1	48.06	49.01	57.35	57.44	62.36	63.03	**65.39**
2	51.16	52.70	62.23	64.98	63.72	74.63	**74.92**
3	50.08	52.23	57.62	56.28	60.45	64.99	**67.40**
4	39.21	40.67	61.26	60.73	67.62	75.28	**78.93**
5	45.77	46.21	49.96	49.12	52.01	58.09	**62.32**
6	42.00	43.16	55.96	56.59	62.82	70.67	**71.89**
7	53.09	54.86	61.69	65.43	66.39	71.53	**73.07**
8	45.18	46.82	57.87	57.87	63.97	65.50	**66.94**
9	49.67	51.14	58.12	55.75	57.65	55.66	**56.42**
10	45.04	45.74	57.85	57.42	60.12	61.75	**65.01**
11	41.03	41.66	46.69	49.38	48.48	51.68	**54.60**
12	39.49	40.17	45.41	44.95	44.74	48.71	**51.14**
13	44.78	44.91	56.93	57.54	51.78	63.54	**64.06**
14	47.22	49.26	51.64	53.32	56.50	59.99	**60.99**
15	49.71	50.78	66.81	67.76	74.04	75.43	**78.66**
Average	46.10	47.29	56.49	56.97	59.51	64.03	**66.12**

**Figure 3:**
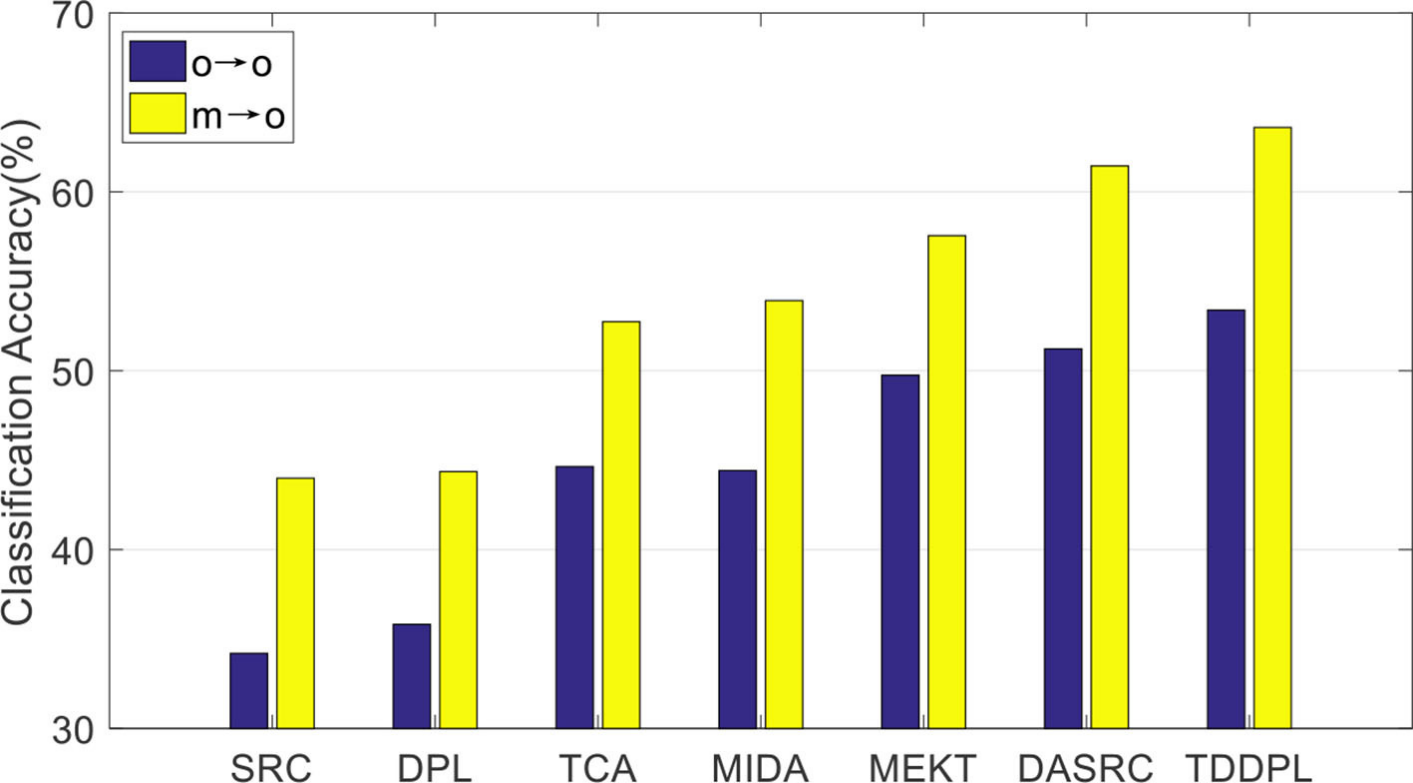
Average classification accuracies on session 2 of the SEED IV dataset.

**Figure 4:**
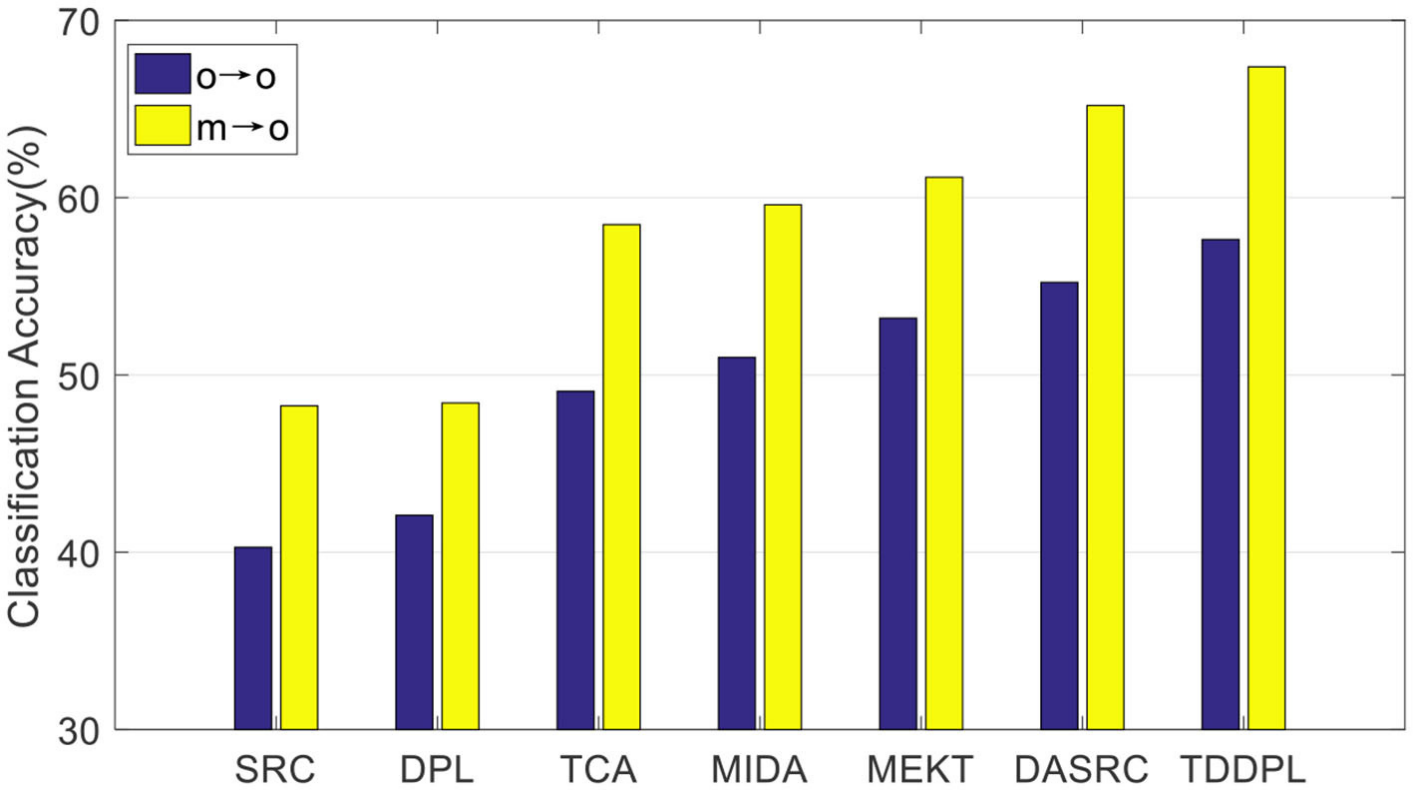
Average classification accuracies on session 3 of the SEED IV dataset.

We compare the average accuracy of the TDDPL approach with other approaches in session 2 and session 3 of the SEED IV dataset, and the average classification accuracies are shown in Figures 3, 4. Similar to the results in Figures 1, 2, the classification performance of the TDDPL approach is the best, indicating that our proposed TDDPL approach can improve the performance of the learning task in the TD by using auxiliary data through dictionary pair learning. We also notice that the performance of all comparison approaches in the o → o scenario is slightly worse than that of m → o scenario of the SEED and SEED IV datasets. This is because in the o → o scenario, when single-subject data is used as the SD, if the correlation between the SD and the TD is low, the effect of transfer learning approach may not be good. In the m → o scenario, multiple SDs expand the capacity of the training set, which can also make the performance of transfer learning approach more stable.

## Conclusion

Aiming at the problem of subject differences in the data distribution of EEG signals; simultaneously, due to insufficient information in the new domain in EEG emotion classification, this study studies how to mine knowledge from other related domains and transfer it to improve the performance of the task in TD. To this end, we propose a transfer discriminative dictionary pair learning approach based on subspace transfer learning. In the subspace, the distribution of TD is similar to that of the SD, and we learn a shared dictionary pair with discriminative ability. The label information of the SD is used to construct a classifier in the TD. However, EEG emotion classification based on transfer learning still faces many challenges. In the following research work, we will focus on the following aspects: (1) The TDDPL approach proposed in this paper is a batch learning mode with high time complexity and is not adopted used in online learning situations. How to improve the training efficiency of TDDPL and propose an online learning approach is an important topic to be studied urgently. (2) The TDDPL approach mainly studies the problem that the SD and TD data and label space are consistent, and it does not consider the situation that the TD label space is unavailable. In this case, how to construct an effective subspace, mine the similarities and differences between domains, and conduct effective knowledge transfer is a direction for future research. (3) This paper studies the homogeneous transfer learning problem, that is, the EEG feature space of the SD is the same as the TD. The problem becomes complicated when the feature spaces differ between domains. So far, the research based on heterogeneous transfer learning is not sufficient. How to use heterogeneous domain knowledge to improve the learning ability of the TD is an important content that needs to be studied in the future.

### Model Parameter Analysis

**Figure 5:**
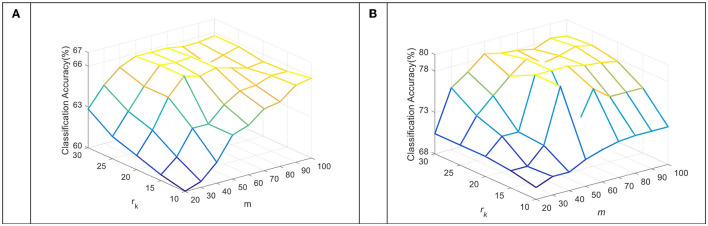
The average classification accuracy of the TDDPL approach under varying *m* and *r*_*k*_ on the SEED dataset. **(A)** o → o scenario, **(B)** m → o scenario.

**Figure 6:**
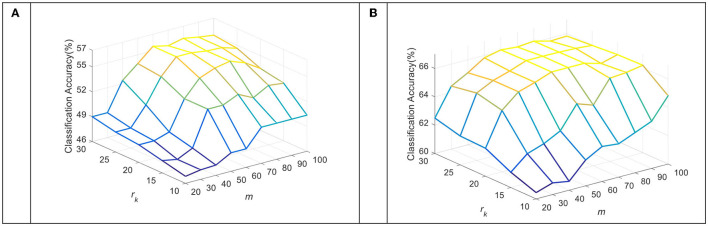
The average classification accuracy of the TDDPL approach under varying *m* and *r*_*k*_ on the SEED IV dataset. **(A)** o → o scenario, **(B)** m → o scenario.

The dimension of the subspace *m* and the number of sub-dictionary atoms *r*_*k*_ per class are key parameters in the TDDPL approach. Figures 5, 6 show the average classification accuracy of the TDDPL approach with varying *m* and. Figure 5 shows the average classification accuracy of o → o and m → o scenarios on session 1 of the SEED dataset. Figure 6 shows the average classification accuracy of the two transfer learning scenarios on session 1 of the SEED IV dataset. It can be seen that the TDDPL approach is optimal when the subspace dimension is about 50 and the number of sub-dictionary atoms per class is about 20 in the SEED dataset. The TDDPL approach is optimal when the subspace dimension is 60 and the number of sub-dictionary atoms per class is about 25 in the SEED IV dataset. In addition, the dimension of the subspace is much smaller than that of the original space, indicating that domain-invariant knowledge can exist in low-dimensional subspaces. The low-dimensional subspace also reduces the training burden of the TDDPL approach.

**Figure 7:**
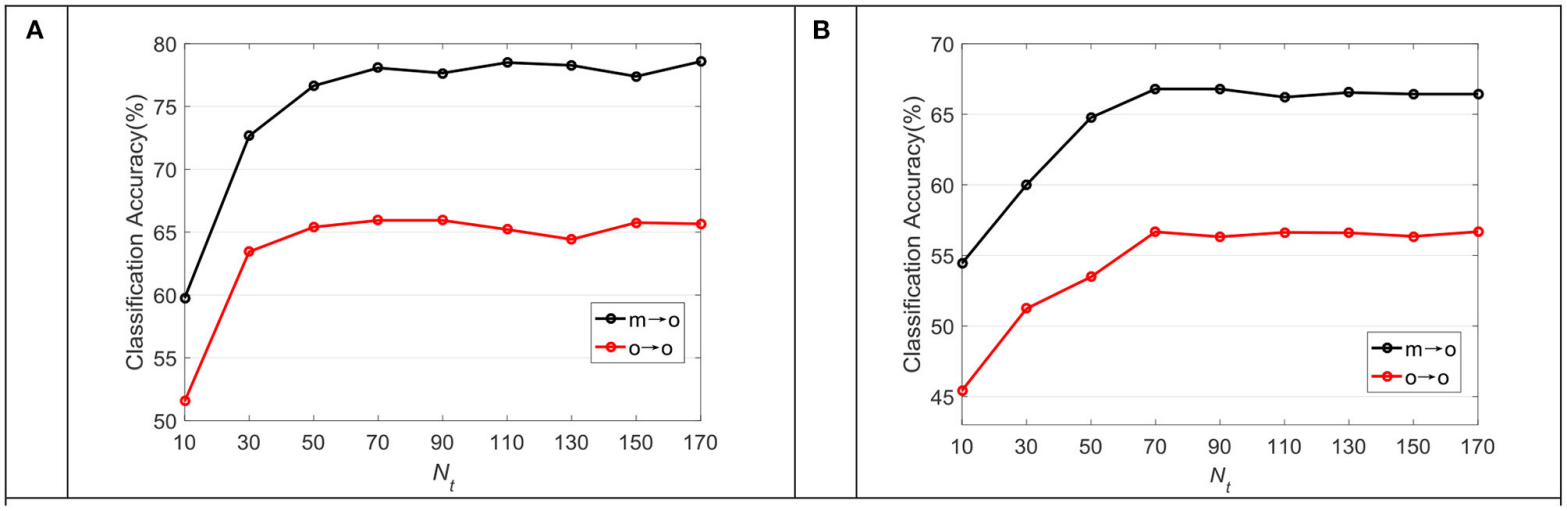
The average classification accuracy of the TDDPL approach under varying *N*_*t*_ in the TD on **(A)** SEED dataset, **(B)** SEED IV dataset.

Figure 7 shows the average classification accuracy of TDDPL under varying training samples in the TD in the o → o and m → o scenarios of the SEED and SEED IV datasets. As can be seen from Figure 7A, the classification performance of our approach is slightly improved with the increase of training samples in the TD, but its improvement speed is very slow. After reaching the highest accuracy, it is not very sensitive to the increase of training samples in TD. We can draw similar observations from Figure 7B. It shows that the TDDPL approach only needs a small number of training samples in the TD to establish the transfer learning model, and obtains the discriminative knowledge from SD.

## Data Availability Statement

Publicly available datasets were analyzed in this study. Public available SEED and SEED IV datasets used in this paper can be found in the following links: https://paperswithcode.com/dataset/seed-1, https://bcmi.sjtu.edu.cn/~seed/downloads.html.

## Author Contributions

TN developed the theoretical framework and model in this study. YR and MD implemented the experiment. TN and YR wrote the manuscript. All authors read and approved the manuscript.

## Funding

This work was supported in part by the Natural Science Foundation of Jiangsu Province under Grant Bk202101333.

## Conflict of Interest

The authors declare that the research was conducted in the absence of any commercial or financial relationships that could be construed as a potential conflict of interest.

## Publisher's Note

All claims expressed in this article are solely those of the authors and do not necessarily represent those of their affiliated organizations, or those of the publisher, the editors and the reviewers. Any product that may be evaluated in this article, or claim that may be made by its manufacturer, is not guaranteed or endorsed by the publisher.

## References

[B1] Ameri R. Pouyan A. Abolghasemi V. (2016). Projective dictionary pair learning for EEG signal classification in brain computer interface applications. Neurocomputing. 218, 382–389, 10.1016/j.neucom.2016.08.082

[B2] Atkinson J. Campos D. (2016). Improving BCI-based emotion recognition by combining EEG feature selection and kernel classifiers. Expert Syst. Appl. 47, 35–41, 10.1016/j.eswa.2015.10.049

[B3] Barthélemy Q. Gouy-Pailler C. Isaac Y. Souloumiac A. Larue A. Mars J. I. (2013). Multivariate temporal dictionary learning for EEG. J. Neurosci. Methods. 215, 19–28, 10.1016/j.jneumeth.2013.02.001 23428648

[B4] Beyeler M. Rounds E. L. Carlson K. D. Dutt N. Krichmar J. L. (2019). Neural correlates of sparse coding and dimensionality reduction. PLoS Comput. Biol. 16, e10069008, 10.1371/journal.pcbi.1006908 31246948 PMC6597036

[B5] Chaplin T. M. (2015). Gender and emotion expression: a developmental contextual perspective. Emotion Rev. 7, 14–21. 10.1177/1754073914544408 26089983 PMC4469291

[B6] Ding Z. Fu Y. (2019). Deep transfer low-rank coding for cross-domain learning. IEEE Trans. Neural Netw. Learn. Syst. 30, 1768–1779. 10.1109/TNNLS.2018.2874567 30371396

[B7] Du H. Zhang Y. Ma L. Zhang F. (2021). Structured discriminant analysis dictionary learning for pattern classification. Knowl. Based Syst. 216, 106794. 10.1016/j.knosys.2021.106794

[B8] Gu X. Fan Y. Zhou J. (2021). Optimized projection and fisher discriminative dictionary learning for EEG emotion recognition. Front. Psychol. 12, 705528, 10.3389/fpsyg.2021.705528 34262515 PMC8274488

[B9] Jiang Z. Lin Z. Davis L. (2013). Label consistent K-SVD: learning a discriminative dictionary for recognition. IEEE PAMI. 35, 2651–2664, 10.1109/TPAMI.2013.88 24051726

[B10] Lan Z. Olga S. Wang L. Reinhold S. Muller-Putz G. R. (2018). Domain adaptation techniques for EEG-based emotion recognition: a comparative study on two public datasets. IEEE Trans. Cogn. Develop. Syst. 11, 85–94, 10.1109/TCDS.2018.2826840

[B11] Li J. Qiu S. Shen Y. Y. Liu C. L. He H. (2020). Multisource transfer learning for cross-subject EEG emotion recognition. IEEE Trans Cybern. 50, 3281–3293, 10.1109/TCYB.2019.2904052 30932860

[B12] Li P. Liu H. Si Y. Li C. Li F. Zhu X.. (2019). EEG based emotion recognition by combining functional connectivity network and local activations. IEEE Trans Biomed Eng. 66, 2869–2881. 10.1109/tbme.2019.2897651 30735981

[B13] Lin Y. P. Tzyy-Ping J. (2017). Improving EEG-based emotion classification using conditional transfer learning. Front. Human Neurosci. 6, 00334, 10.3389/fnhum.2017.00334 28701938 PMC5486154

[B14] Mert A. Akan A. (2018). Emotion recognition from EEG signals by using multivariate empirical mode decomposition. Pattern Anal. Appl. 21, 81–89. 10.1007/s10044-016-0567-6

[B15] Morioka H. Kanemura A. Hirayama J. Shikauchi M. Ogawa T. Ikeda S.. (2015). Learning a common dictionary for subject-transfer decoding with resting calibration. NeuroImage. 111, 167–178. 10.1016/j.neuroimage.2015.02.015 25682943

[B16] Ni T. Ni Y. Xue J. Wang S. (2021). A domain adaptation sparse representation classifier for cross-domain electroencephalogram-based emotion classification. Front. Psychol. 12, 721266, 10.3389/fpsyg.2021.721266 34393958 PMC8358659

[B17] Pan S. J. Tsang I. W. Kwok J. T. Yang Q. (2011). Domain adaptation via transfer component analysis. IEEE Trans. Neural Netw. 22, 199–210, 10.1109/TNN.2010.2091281 21095864

[B18] Ramakrishnan A. G. Panachakel J. T. (2021). Decoding covert speech from EEG-a comprehensive review. Front. Neurosci. 4, 642251, 10.3389/fnins.2021.642251 33994922 PMC8116487

[B19] Sheykhivand S. Rezaii T. Y. Mousavi Z. Delpak A. Farzamnia A. (2020). Automatic identification of epileptic seizures from EEG signals using sparse representation-based classification. IEEE Access. 8, 138834–138845, 10.1109/ACCESS.2020.3011877

[B20] Shu L. Xie J. Yang M. Li Z. Li Z. Liao D.. (2018). A review of emotion recognition using physiological signals. Sensors. 18, 2074. 10.3390/s18072074 29958457 PMC6069143

[B21] Sorkhabi M. M. (2014). Emotion detection from EEG signals with continuous wavelet analyzing. Psychophysiology. 38, 912–925, 10.1111/1469-8986.3860912 12240668

[B22] Wan Z. Yang R. Huang M. Zeng N. Liu X. (2021). A review on transfer learning in EEG signal analysis. Neurocomput. 421, 1–14, 10.1016/j.neucom.2020.09.017

[B23] Wang J. Guo Y. Guo J. Li M. Kong X. (2017). Synthesis linear classifier based analysis dictionary learning for pattern classification. Neurocomputing. 238, 103–113, 10.1016/j.neucom.2017.01.041

[B24] Wright J. Yang A. Y. Ganesh A. Sastry S. S. Ma Y. (2009). Robust face recognition via sparse representation. IEEE PAMI. 31, 210–227, 10.1109/TPAMI.2008.79 19110489

[B25] Yan K. Kou L. Zhang D. (2018). Learning domain-invariant subspace using domain features and independence maximization. IEEE Trans Cybern. 48, 288–299, 10.1109/TCYB.2016.2633306 28092587

[B26] Zanini P. Congedo M. Jutten C. Said S. Berthoumieu Y. (2018). Transfer learning: a riemannian geometry framework with applications to brain-computer interfaces. IEEE Trans. Biomed. Engineer. 65, 1107–1116, 10.1109/TBME.2017.2742541 28841546

[B27] Zhang Q. Lee M. (2009). Analysis of positive and negative emotions in natural scene using brain activity and GIST. Neurocomputing. 72, 1302–1306. 10.1016/j.neucom.2008.11.007

[B28] Zhang W. Wu D. (2020). Manifold embedded knowledge transfer for brain-computer interfaces. IEEE Trans. Neural Syst. Rehabilitation Eng. 28, 1117–1127. 10.1109/TNSRE.2020.2985996 32286993

[B29] Zhang Z. Jiang W. Qin J. Zhang L. Li F. Zhang M.. (2018). Jointly learning structured analysis discriminative dictionary and analysis multiclass classifier. IEEE Trans. Neural Netw Learn Syst. 29, 3798–3814, 10.1109/TNNLS.2017.2740224 28922127

[B30] Zheng W. Liu W. Lu Y. Cichocki A. B. L. (2019). Emotionmeter: a multimodal framework for recognizing human emotions. IEEE Trans Cybern. 49, 1110–1122, 10.1109/TCYB.2018.2797176 29994384

[B31] Zheng W. Lu B. L. (2015). Investigating critical frequency bands and channels for EEG-based emotion recognition with deep neural networks. IEEE Trans. Autonomous Mental Dev. 7, 162–175. 10.1109/TAMD.2015.2431497

[B32] Zhu J. Shen Z. Ni T. (2022). Multi-frequent band collaborative EEG emotion classification method based on optimal projection and shared dictionary learning. Front. Aging Neurosci. 2, 848511, 10.3389/fnagi.2022.848511 35250551 PMC8892240

[B33] Zhuang N. Ying Z. Tong L. Chi Z. Zhang H. Yan B. (2017). Emotion recognition from EEG signals using multidimensional information in EMD domain. BioMed Res. Int. 2017, 8317357, 10.1155/2017/8317357 28900626 PMC5576397

